# A Distance Bounding Protocol for Location-Cloaked Applications

**DOI:** 10.3390/s18051337

**Published:** 2018-04-26

**Authors:** Cristián Molina-Martínez, Patricio Galdames, Cristian Duran-Faundez

**Affiliations:** 1 Magister en Ciencias de la Computación, Universidad del Bío-Bío, Concepción 4051381, Chile; crmolina@ubiobio.cl; 2 Departamento de Ciencias de la Computación, Universidad del Bío-Bío, Concepción 4051381, Chile; 3 Departamento de Ingeniería Eléctrica y Electrónica, Universidad del Bío-Bío, Concepción 4051381, Chile; crduran@ubiobio.cl

**Keywords:** location verification, location privacy, location refinement, wireless ad-hoc networks

## Abstract

Location-based services (LBSs) assume that users are willing to release trustworthy and useful details about their whereabouts. However, many location privacy concerns have arisen. For location privacy protection, several algorithms build a cloaking region to hide a user’s location. However, many applications may not operate adequately on cloaked locations. For example, a traditional distance bounding protocol (DBP)—which is run by two nodes called the prover and the verifier—may conclude an untight and useless distance between these two entities. An LBS (verifier) may use this distance as a metric of usefulness and trustworthiness of the location claimed by the user (prover). However, we show that if a tight distance is desired, traditional DBP can refine a user’s cloaked location and compromise its location privacy. To find a proper balance, we propose a location-privacy-aware DBP protocol. Our solution consists of adding some small delays before submitting any user’s response. We show that several issues arise when a certain delay is chosen, and we propose some solutions. The effectiveness of our techniques in balancing location refinement and utility is demonstrated through simulation.

## 1. Introduction

Distance bounding (DB) is the process that allows an entity called a *verifier* to estimate a tight distance from its location to the location of a second entity called the *prover*. Distance bounding can become an important cornerstone to face many access control problems requiring not only the verification of a user’s credentials but also its location (e.g., unlocking an automobile door or igniting a car engine). Here, the prover—who has a car key or token—needs to be close enough to the car lock in order to unlock it [1]. Other applications, such as location-based routing, location-aided routing (LAR) [2], distance routing effect algorithm for mobility (DREAM) [3], and greedy perimeter stateless routing (GPSR) [4] may be enhanced with DB. These protocols assume that mobile nodes are acting honestly and are forwarding messages only when they can get messages closer to their destination. Finally, other examples are location-based services (LBSs), which rely on the trustworthiness of the location provided by their users. Particularly, new companies like Placecast [5] have focused their businesses on providing location verification services for location-based marketing companies. Placecast claims that more than 25% of the location-based advertisements are targeted improperly.

However, several works have highlighted the problem of invading a user’s privacy, since location information can be used to infer the user’s lifestyle and can present significant privacy and safety threats. This problem has actually motivated a series of research on location cloaking [6,7,8,9,10,11,12,13,14]. The key idea of these proposed techniques is to reduce location resolution to achieve a desired level of protection. Instead of its precise location, a node discloses a geographic region as its location. This region, referred to as a cloaking region, contains the node’s current position and needs to satisfy other constraints, depending on the risks of concern.

A cloaking region must guarantee that its owner can be located at any position with similar likelihood. For example, for anonymous uses of location-based services (e.g., [6,7,8]), a cloaking region needs to contain at least *K* different nodes; for location privacy protection (e.g., [9,10,13,14]), a cloaking region must be visited by *K* different users at different times; for location safety protection ([11,12]), node density in a cloaking region must not exceed some threshold.

However, the use of cloaked locations presents many challenges to most of the existing location-based applications. These services are often designed under the assumptions that the user (prover) is willing to (1) disclose its exact location and (2) allow the service provider (verifier) to localize its position as precisely as possible. These assumptions may not hold in reality for traditional distance bounding protocol. A DBP tries to achieve a tight bounding distance between the prover and verifier, and we show that this goal can potentially refine a node’s cloaked location.

The problem of distance bounding in wireless networks was initially investigated in [15,16] without taking cloaked locations into account. In these works, there are two nodes—the verifier (*V*) and the prover (*P*)—exchanging a series of challenges and responses. Based on the Radio Frequency (RF) propagation delay, the verifier computes a tight circular region containing the prover’s exact position. This result can be used by *V* to accept a location claimed by *P* only if such a location is within this tight circular region. These initial works also assume that the communication channel between *P* and *V* is reliable, meaning that all messages arrive to their destination. However, authors in [17,18] study the challenges faced in building a DB protocol when a lossy and noisy communication channel is present.

The DB problem has also been extended to support multiple provers (known as group distance bounding) [19] and also to support multiple verifiers (known as secure positioning) [20]. In this latter scenario, multiple verifiers are used to further narrow down the area where the prover is located. However in this article, we are considering the original scenario where a single verifier and prover are present, and a reliable channel is assumed. Thus, we can only achieve distance bounding but not secure positioning. Moreover, in this paper we assume a wireless propagation radio based on a binary disk model. That is, an ideal communication channel with circular covering ranges, completely reliable communication (so, no losses inside the covering range), and propagation times exclusively depending on the linear distance between the source and the destination.

In this paper, we consider the problem of preventing leaking location details about the prover when this node runs a DB protocol against only one verifier node. *P* claims to be inside a cloaking region, but *V*’s location is unknown. Node *P* is willing to run a DB protocol while providing a certain level of guarantee that its cloaking region will not be refined during the DB process. We consider a cloaking region as refined if the adversary (e.g., the verifier) can conclude that *P* must be in some region whose entropy is smaller than the one provided by its cloaking region. We present a location refinement attack, named as distance bounding attack (DBA). In this attack, *V* refines a cloaking region by measuring the round trip time of its communication with the prover.

An initial idea to tackle this problem is to let node *P* delay any message from *V* on purpose to seem to be further away. However, this approach has at least two drawbacks. Firstly, if node *V* knows some lower or upper bound of such a delay, then it can adjust the distance computed from the DBA and refine *P*’s cloaking region. Secondly, if such a delay is random but too large, node *V* may estimate a useless distance bounding for any location-based application. To overcome the aforementioned drawbacks, we propose a location-privacy-aware distance bounding protocol that limits the likelihood a cloaked location is refined. We show its effectiveness against DBA by measuring the number of successful location refinements when *P* and *V* run our protocol.

To the best of our knowledge, the state-of-the-art research on traditional DB assume some of the following conditions [21,22,23,24,25,26]. Node *P* is willing to disclose its exact location to *V*, and the adversary is a third party eavesdropping the communication between *P* and *V*. These works [21,22] show that a location-privacy leakage can arise when the adversary is able to infer location details about the prover and the verifier from the traffic. Moreover, authors in [22] show that it is not possible to prevent location-privacy leakage in current DB protocols when multiple third parties are colluded and eavesdropping the traffic between *P* and *V*. However, the same authors claim that such a risk of leakage can at least be mitigated. Finally, other works [23,24,25,26] assume the same adversary model but they analyze DB protocols against a third party performing a man-in-the-middle attack (i.e., mafia, terrorist, or distance hijacking attacks).

However, our work is based on different assumptions and restrictions. First, node *P* is not willing to disclose an exact location but a cloaking region instead. Second, node *P* is willing to accept some loss of location-privacy only if this leakage is not greater than a node *P*’s threshold. Third, our adversary model assumes that our attacker is the same node *V* who wants to obtain more details about *P*’s whereabout. Lastly, we are addressing the distance bounding attack (DBA), which is different from those currently man-in-the-middle attacks studied in the literature.

The remainder of this paper is organized as follows. We discuss DBA in detail and present their solutions in Section 2. The proposed distance bounding protocol is presented in Section 3. We evaluate the effectiveness of our solution against DBA in Section 4. Finally, we offer concluding remarks in Section 5.

## 2. System Overview

We initially assume that there are two stationary nodes called the prover (*P*) and the verifier (*V*). Node *P* is honest and does not know any details about node *V*’s whereabouts. However, the location of *P* is represented by a public cloaking region ($R_{pub}$). This region includes *P*’s exact location and must satisfy the location privacy and/or safety requirements demanded by *P*. $R_{pub}$ can have any shape, but without loss of generality we assume that it is a circle centered at some point $O_{pub}$ and radius $r_{pub}$. Many techniques have been proposed to compute a cloaking region [12,13,14,27,28]. Our work does not assume that some specific technique should be used, but for the sake of simplicity we assume that there exists a trustworthy anonymizer (AS) in charge of computing cloaking regions.

Node *P* claims to be at a certain location ($R_{pub}$) and node *V* runs a distance bounding protocol to validate this claim. We assume that node *P* is willing to participate in this process, but it does not want node *V*—the adversary—to increase the resolution of its location. We say that *P*’s location has been refined if node *V* can conclude there exists a sub-region where *P* must be located whose entropy is smaller than the entropy of $R_{pub}$. An entropy of a cloaking region can be computed as suggested by Nu et al. [13].

Each node is equipped with an RF network interface operating with only one omni-directional antenna. An interface is used to either broadcast or receive a message. Due to the characteristics of this antenna, neither node *P* nor node *V* are able to determine the direction of an incoming message. Finally, we assume there is no collusion among nodes and a node’s processing time can be negligible with respect to the propagation time. Rasmussen et al. [29] design a network interface for DB which can achieve processing time smaller than one nanosecond.

Although the goal of this paper is not to prevent location-privacy leakage when a third party is colluded with others, many complimentary techniques can be used to mitigate this attack. For example, either using low-power transmission to reduce the region where a message can be listened [30], or using any scheme based on either FHSS (frequency-hopping spread spectrum) [31] or DSSS [21] (direct sequence spread spectrum). By using FHSS, the prover and the verifier can select a secret and pseudo random sequence of frequency channels beforehand. When a DB protocol is run, *V* and *P* can switch between these channels to prevent interception. Another alternative is by using DSSS, since any signal transmitted either by the prover or the verifier is spread over a large frequency band, then any third party might not been able to distinguish it from the channel background noise.

### 2.1. A Distance Bounding Attack

Suppose that nodes *V* and *P* exchange a series of challenge–response messages. Every time *P* replies immediately to a challenge, it may put its location privacy at risk. Node *V* measures the round-trip time between a challenge and its response, and concludes with an upper-bound on the distance to *P*. This upper-bound distance (${\tilde{d}}_{VPmax}$) is computed as half of the average round trip time multiplied by the speed of light. To conclude this attack, *V* overlaps a distance bounding circle, which is a circle centered at its position and radius ${\tilde{d}}_{VPmax}$, with *P*’s cloaking region, and concludes with more details about *P*’s location.

**Figure 1:**
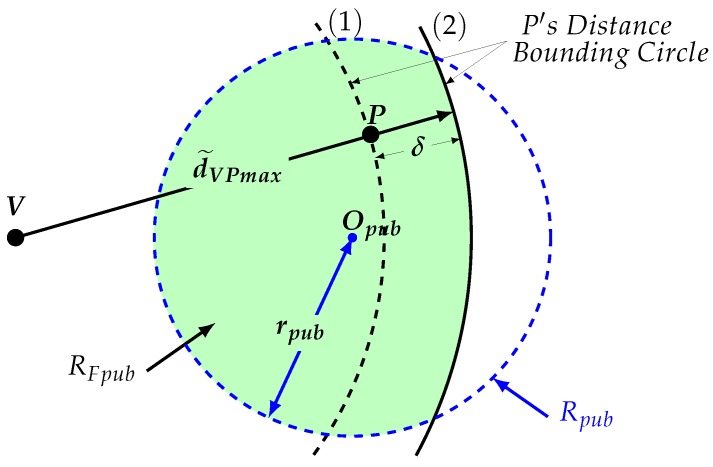
A distance bounding attack (DBA).

For example, consider the scenario represented by Figure 1. In this case, *P* replies immediately to a challenge sent from *V*, and this latter node can conclude that all possible locations for *P* are all points over the perimeter of *V*’s distance bounding circle (1) that are also within *P*’s cloaking region. If *P* had added an intentional delay (δ) to look more distant, then *V* would have computed a longer distance bounding circle (2). Since *V* does not know the amount of delay added by *P*, this node can only conclude that all locations within the colored area are the only feasible locations for *P*. We say that node *V* in Figure 1 has been able to refine node *P*’s location if the entropy of $R_{Fpub}$ is smaller than the entropy of $R_{pub}$.

### 2.2. Thwarting a DBA

Since *P* wants to prevent location refinement when receiving a challenge from *V*, it can broadcast any reply only after having waited for a short time. There are many ways to choose this delay. One approach is to choose it to be equal to the maximum distance from *V* to the perimeter of $R_{pub}$ ($d_{max}$). *P* might think it can defeat DBA, however, since $d_{max}\geq r_{pub}$, then *V* might still be able to refine $R_{pub}$ by subtracting $r_{pub}$ from ${\tilde{d}}_{VPmax}$.

Another alternative way is to allow node *P* to uniformly choose a secret random delay from an interval [0,Λ]. Galdames et al. [32] proved that Λ can be computed as a function of the maximum probability of refinement tolerated by *P*. However, the closer to zero this probability is, the larger ${\tilde{d}}_{VPmax}$ becomes. If node *V* demands a tight upper-bound for ${\tilde{d}}_{VPmax}$, it can happen that *P*’s maximum probability of refinement may not be achieved.

Our idea to overcome the aforementioned drawbacks is to allow node *P* to request to the AS a second cloaking region denoted as $R_{priv}$. Node *P* must demand from the AS that knowledge of $R_{pub}$ does not release any clue about the location and shape of $R_{priv}$. The only known fact is that $R_{priv}$ is entirely located within $R_{pub}$. Without loss of generality, we assume $R_{priv}$ is a circle centered at some point $O_{priv}$ and radius $r_{priv}$.

Now, we say that *P*’s location has not been compromised or refined if and only if $R_{Fpub}$ and $R_{priv}$ have similar location anonymity and privacy features. Formally, we say that node *P*’s location has been successfully refined if and only if the entropy of $R_{Fpub}$ is smaller than the entropy of $R_{priv}$.

In summary, a suitable location-privacy-aware DBP must ensure it completely covers $R_{priv}$ and gives the minimum possible information to *V*, so $R_{priv}$ cannot be refined.

## 3. Protocol Proposal

For our protocol, we propose the use of two cloaking regions: First, a private region $R_{priv}$, which will be only known by *P*; and second, a public region $R_{pub}$ which is sent from *P* to *V* as its claimed location. Additionally, the answering time of *P* to the challenge of *V* is modified by adding a certain delay δ selected in an interval such that $d_{\left(\delta \right)}\in \left[d_{maxpriv},2r_{priv}\right]$, where $d_{\left(\delta \right)}$ is the distance traveled by the electromagnetic signal at time δ, $d_{maxpriv}$ denotes the longest distance between *P* and $R_{priv}$’s perimeter, and $r_{priv}$ is $R_{priv}$’s radius. Node *V* runs a DBA against *P* to verify *P*’s claimed position.

**Figure 2:**
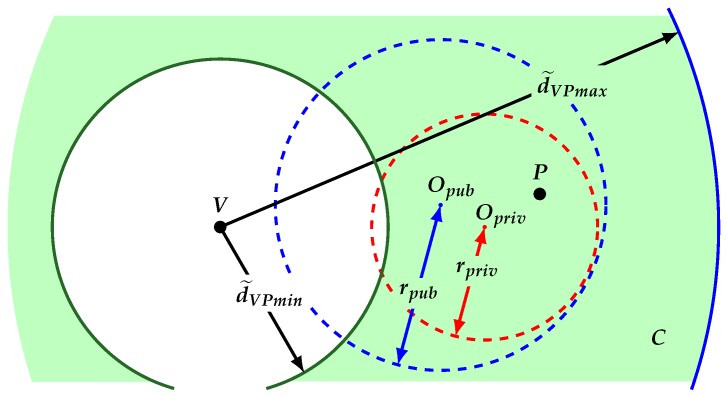
Attack of refinement that determines the region *C*, color region, in which *P* and the region $R_{Fpub}$, color region within the major circumference, must be found.

As is exemplified in Figure 2, simple modifications to DBA allow *V* to determine a region *C* consisting of a ring-like region centered at *V* and with width equal to $R_{pub}$’s diameter, within which *P* should be located. By intersecting *C* and $R_{pub}$, a first level of refining can be performed, determining a (refined) region $R_{Fpub}$ where *P* should be located.

### 3.1. Metrics

The participation of *P* in the protocol is subject to the level of refinement of region *R*, which must be evaluated, and should not exceed a given tolerance factor τ. To that, we define the metric $\Upsilon_{H}$ as the ratio between the entropy of $R_{Fpub}$ and the entropy of $R_{priv}$, as described by Equation (1):

(1) $$\Upsilon_{H}=\frac{H(R_{Fpub})}{H(R_{priv})}.$$

For simplification purposes, said metric is simplified to the ratio between the areas of $R_{Fpub}$ and $R_{priv}$, as the probability of *P* being in any place of $R_{priv}$ is considered equally likely. Since *P* does not know the location of *V*, it assumes the worst situation of refinement to decide its participation in the DBP; i.e., *V* is located at the maximum possible distance from *P* on the axis intersecting the position of *P* and the center of $R_{pub}$, with $O_{pub}$ between both nodes.

Node *P* considers $\Upsilon_{H}$, τ, and the condition of maximum possible refinement in deciding whether or not to participate in the protocol; that is, *P* will answer to *V*’s challenge if $\Upsilon_{H}\geq \tau$, as described by Equation (2):

(2) $$\Upsilon_{H}=\frac{Area\;of\;R_{Fpub-min}}{Area\;of\;R_{priv}}\geq \;\tau ,$$

where the Area of $R_{Fpub-min}$ is determined by *P* considering the condition of maximum possible refinement.

### 3.2. Protocol

The determination of the cloaking regions may require some information which is not available to *P*; therefore, we consider that such information is provided by an AS.

**Figure 3:**
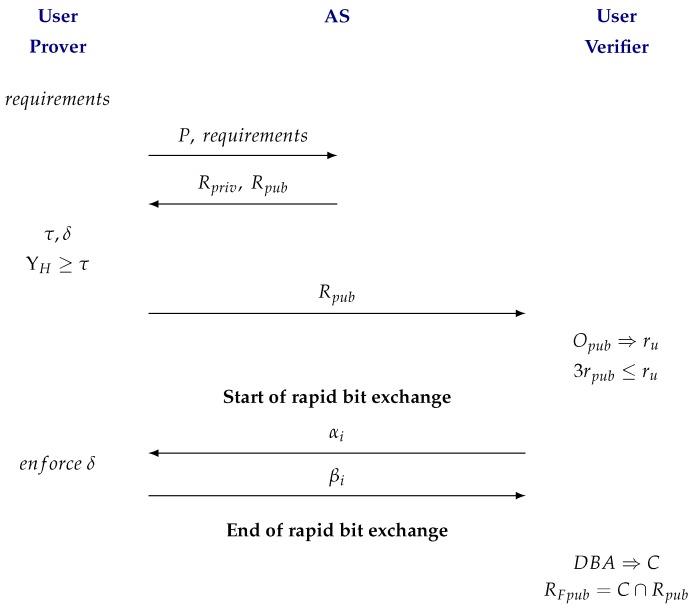
Distance Bounding Protocol Aware of Location Privacy (DBP-ALP) for static users. AS: trustworthy anonymizer.

Given all of the above, we introduce the Distance Bounding Protocol Aware of Location Privacy (DBP-ALP) for static users, which is summarized in Figure 3 and considers DBA as a procedure for *V* to estimate its distance to *P*.

1. User *P* determines the privacy and security criteria for the cloaking regions $R_{priv}$ and $R_{pub}$. With those, it asks the AS entity to create both regions.
2. The AS answers *P* with the regions $R_{priv}$ and $R_{pub}$.
3. User *P* determines the refinement level τ.
4. With the information of regions $R_{priv}$ y $R_{pub}$, user *P* randomly selects the distance which defines the delay δ on the interval $\left[d_{maxpriv},2r_{priv}\right]$.
5. For the case of maximum possible refinement, user *P* decides to continue participating with *V* if condition $\Upsilon_{H}\left(P,\delta ,R_{priv},R_{pub}\right)\geq \tau$ is satisfied.
6. If the protocol is continued, *P* sends region $R_{pub}$ to *V*.
7. *V* determines if region $R_{pub}$ satisfies a practical minimum precision, and decides if it will continue with the protocol.
8. Through a DBA, *V* determines region *C*. During that process, *P* delays its answer by a given time δ.
9. *V* determines the region were *P* must be by intersecting regions $R_{pub}$ and *C*.

## 4. Results and Discussion

The proposed protocol is compared against several baseline and improved approaches:

1. *Solution with a delay equal to maximal distance* (SMD), which is implemented with a cloaking region and a delay based on the maximum distance within the cloaking region.
2. *Solution with an interval of possible delays* (SID), which is considered a cloaking region and a delay chosen at random from ([0,Λ]).
3. *Solution with a delay equal to maximal distance with double cloaking regions* (SMD-DCR), where two cloaking regions are considered and a delay based on the maximum distance within $R_{priv}$.
4. *Solution with an interval of possible delays with double cloaking regions* (SID-DCR), where two cloaking regions are considered and a selected delay chosen at random from ($d_{\left(\delta \right)}\;\in \;\left[d_{maxpriv},2r_{priv}\right]$).
5. *Solution with an interval of possible delays with double cloaking regions and entropy usage* (SID-DCR-E); it considers a location privacy leakage only if the entropy of the refined regions is smaller than the entropy of $R_{priv}$.

**Figure 4:**
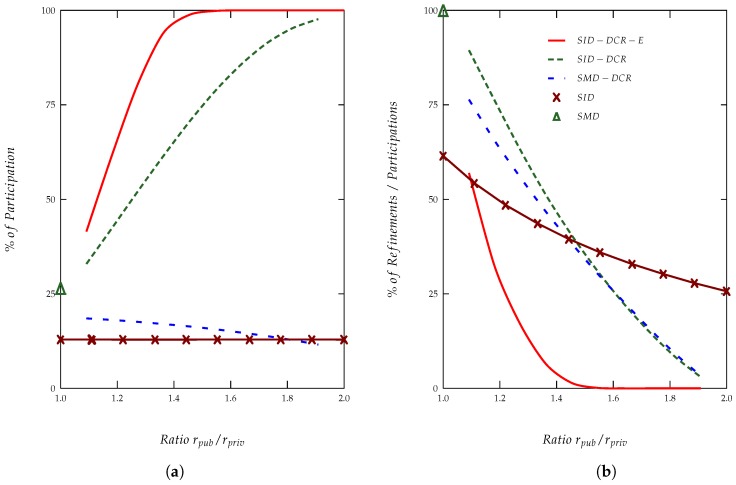
(**a**) Percentage of full participations and (**b**) percentage of refinements over the number of full participations in the DBP-ALP. SID: solution with an interval of possible delays; SID-DCR: solution with an interval of possible delays with double cloaking regions; SID-DCR-E: solution with an interval of possible delays with double cloaking regions and entropy usage; SMD: solution with a delay equal to maximal distance; SMD-DCR: solution with a delay equal to maximal distance with double cloaking regions.

The refinement conditions for the proposed protocol, along with the other studied solutions, have been implemented and simulated. Simulation results, considering both users *P* and *V* as static entities and a level of refinement, are presented in Figure 4, SMD-DCR presents a second level of refinement, as it delivers information by selecting the delay over the maximum distance. The graphic shows (1) the percentages of participation in the entire protocol (i.e., when both users participate in the rapid bit exchange) and (2) the percentages of times in which there is a refinement; both of them are functions of the ratio $r_{pub}/r_{priv}$.

As shown in Figure 4, we can observe the following characteristics of the proposed protocol: (1) it provides full protection (i.e., there is no possible refinement) against refinement attempts when is given that $r_{pub}\geq 2r_{priv}$; and (2) when it is given that $\sqrt{2}\leq \left(r_{pub}/r_{priv}\right)<2$, such a protection presents an average participation greater than 96%, along with a low percentage of refinement cases (under 5%). In both cases, the maximum possible refinement agrees with the tolerance factor τ defined by *P*.

## 5. Conclusions

This paper introduces a Distance Bounding Protocol Aware of the Location Privacy (DBP-ALP). The protocol allows participation in a distance bounding process between a prover *P* and a verifier *V*, along with consideration of a cloaking region protected against DBA refinements.

DBP-ALP allows both users *P* and *V* to leave the protocol execution if their participation conditions are not satisfied. On one hand, *P* may exit the protocol if its maximum refinement restriction is not satisfied. Such a decision is proposed to consider a metric concerning the relation between the entropies of the involved regions. On the other hand, *V* may leave the protocol if the cloaking region provided by *P* exceeds a usefulness size.

From the results, DBP-ALP provides full protection against refinement attempts when the radius of the public region exceeds twice the private region radius. Besides, participation average of both users are ensured in 96% when the ratio between the public and private radii exceeds $\sqrt{2}$. These results show that DBP-ALP is suitable for protecting the prover location privacy, along with respecting its refinement tolerance and a maximum distance criterion accepted by the verifier.

Our future efforts will be focused on the following points: to minimize the possibility that the user *P* tries to validate a false location and to modify DBP-ALP in order to face a new adversary model consisting of a third party. This new party may perform either a mafia fraud attack or terrorist fraud attack [23,24,25,26]. Finally, a three-dimensional case subject to more real propagation models needs to be considered. Further studies will be focused on this challenging issue, as it represents a more realistic application scenario.

## References

[1] Ford Safe and Secure SecuriCode™ Keyless Entry. https://www.ford.com.pr/en/technology/securicode-keyless-entry-keypad/.

[2] Ko Y., Vaidya N.H. Location-Aided Routing (LAR) Mobile Ad Hoc Networks. Proceedings of the 4th Annual ACM/IEEE International Conference on Mobile Computing and Networking.

[3] Royer E., Perkins C.E. Multicast Operation of the Ad-hoc On-Demand Distance Vector Routing Protocol. Proceedings of the 5th Annual ACM/IEEE International Conference on Mobile Computing and Networking.

[4] Karp B., Kung H.T. GPSR: Greedy Perimeters Stateless Routing for Wireless Network. Proceedings of the 6th Annual International Conference on Mobile Computing and Networking.

[5] Placecast (2017). Location Verification. http://placecast.net/location-verification.html.

[6] Ghinita G., Kalnis P., Skiadopoulos S. PRIVE: Anonymous location-based queries in distributed mobile systems. Proceedings of the 16th International Conference on World Wide Web.

[7] Xu T., Cai Y. Location Anonymity in Continuous Location-based Services. Proceedings of the 15th Annual ACM International Symposium on Advances in Geographic Information Systems.

[8] Hu H., Xu J. Non-Exposure Location Anonymity. Proceedings of the IEEE 25th International Conference on Data Engineering.

[9] Xu T., Cai Y. Exploring Historical Location Data for Anonymity Preservation in Location-based Services. Proceedings of the IEEE 27th Conference on Computer Communications.

[10] Xu T., Cai Y. Feeling-based Location Privacy Protection for Location-based Services. Proceedings of the 16th ACM Conference on Computer and Communications Security.

[11] Xu T., Cai Y. Location Cloaking for Safety Protection of Ad Hoc Networks. Proceedings of the IEEE International Conference on Computer Communications.

[12] Xu T., Cai Y. (2009). Location safety protection in ad hoc networks. Ad Hoc Netw..

[13] Niu B., Li Q., Zhu X., Cao G., Li H. Achieving k-anonymity in Privacy-Aware Location-Based Services. Proceedings of the 33rd Conference on Computer Communications (INFOCOM’14).

[14] Niu B., Gao S., Li F., Li H., Lu Z. Protection of location privacy in continuous LBSs against adversaries with background information. Proceedings of the International Conference on Computing, Networking and Communications (ICNC).

[15] Brands S., Chaum D. (1993). Distance-Bounding Protocols (Extended Abstract). Workshop on the Theory and Application of Cryptographic Techniques (EUROCRYPT’93).

[16] Hancke G.P., Kuhn M.G. An RFID Distance Bounding Protocol. Proceedings of the 1st International Conference on Security and Privacy for Emerging Areas in Communications Networks (SECURECOMM’05).

[17] Singelée D., Preneel B. (2007). Distance bounding in noisy environments. Security and Privacy in Ad-Hoc and Sensor Networks.

[18] Hoda J., Abolfazl F. (2015). Achieving an appropriate security level for distance bounding protocols over a noisy channel. Telecommun. Syst..

[19] Čapkun S., Rasmussen K., Kasper M., Čagalj M., Srivastava M. (2008). Secure Location Verification with Hidden and Mobile Base Stations. IEEE Trans. Mob. Comput..

[20] Sastry N., Shankar U., Wagner D. Secure verification of location claims. Proceedings of the 2nd ACM Workshop on Wireless Security (WiSe’03).

[21] Rasmussen K., Čapkun S. Location privacy of distance bounding protocols. Proceedings of the 15th ACM Conference on Computer and Communications Security.

[22] Mitrokotsa A., Onete C., Vaudenay S. (2014). Location leakage in distance bounding: Why location privacy does not work. Comput. Secur..

[23] Avoine G., Trujillo-Rasua R. (2015). Comparing distance bounding protocols: A critical mission supported by decision theory. Comput. Commun..

[24] Entezari R., Tajamolian M. (2017). RFID unilateral distance bounding protocols: A trade-off between mafia and distance fraud. Comput. Commun..

[25] Zhuang Y., Yang A., Hancke G., Wong D., Yang G. (2017). Energy-Efficient Distance-Bounding with Residual Charge Computation. IEEE Trans. Emerg. Top. Comput..

[26] Pagnin E., Yang A., Hub Q., Hancke G., Mitrokotsa A. (2018). Distance bounding meets human based authentication. Future Gener. Comput. Syst..

[27] Gruteser M., Grunwald D. Anonymous Usage of Location-based Services through Spatial and Temporal Cloaking. Proceedings of the 1st International Conference on Mobile Systems, Applications and Services.

[28] Chow C.Y., Mokbel M., Liu X. A peer-to-peer spatial cloaking algorithm for anonymous location-based service. Proceedings of the 14th Annual ACM International Symposium on Advances in Geographic Information Systems.

[29] Rasmussen K., Čapkun S. Realization of RF distance bounding. Proceedings of the 19th USENIX Conference on Security.

[30] Ranganathan A., Danev B., Capkun S. (2014). Low-power Distance Bounding. arXiv.

[31] Riahi-Manesh M., Kaabouch N. (2017). Analysis of vulnerabilities, attacks, countermeasures and overall risk of the Automatic Dependent Surveillance-Broadcast (ADS-B) system. Int. J. Crit. Infrastruct. Prot..

[32] Galdames P. (2012). Novel Techniques for Location-Cloaked Applications. Ph.D. Thesis.

